# Alginate hydrogel beads embedded with drug-bearing polycaprolactone microspheres for sustained release of paclobutrazol

**DOI:** 10.1038/s41598-021-90338-9

**Published:** 2021-05-25

**Authors:** Alexandra Mun, Haneen Simaan Yameen, Giora Edelbaum, Dror Seliktar

**Affiliations:** 1 Faculty of Biomedical Engineering, Technion Israel Institute of Technology, 32000 Haifa, Israel; 2 Directorate of Defense Research & Development, IDF, Tel Aviv, Israel

**Keywords:** Drug delivery, Field trials

## Abstract

In recent years there has been a growing demand for the development of agrochemical controlled release (CR) technologies. In the present study, we aimed to create a novel agricultural CR device using two polymeric systems that have been predominantly employed in biomedical applications: beads of alginate hydrogel embedded with drug-bearing Polycaprolactone (PCL) microspheres. The combined device utilizes the advantages of each polymer type for biodegradation and controlled release of Paclobutrazol (PBZ), a common growth retardant in plants. Surface morphology of the alginate beads was characterized by scanning electron microscopy (SEM) and water immersion tests were performed for stability and controlled release measurements. Bioassays were performed both in accelerated laboratory conditions and in field conditions. The results showed a capability to control the size of PBZ-loaded PCL microspheres through modification of homogenization speed and emulsifier concentration. Enlargement of PCL microsphere size had an adverse effect on release of PBZ from the alginate device. The growth of oatmeal plants as a model system was affected by the controlled release of PBZ. The preliminary field experiment observed growth retardation during two consecutive rainy seasons, with results indicating a substantial benefit of the sustained growth inhibition through the controlled release formulation. The final product has the potential to be used as a carrier for different substances in the agrochemical industry.

## Introduction

Controlled release (CR) is defined as: “the permeation-regulated transfer of an active ingredient (AI) from a reservoir to a targeted surface to maintain a predetermined concentration level for a specified period of time”^1^. CR has been applied in the agricultural, biomedical, food and pharmaceutical industries to deliver pesticides, herbicides, fertilizers, biomolecules, and drugs^2–5^. The development of CR devices for agriculture have culminated in a number of successful products. These agricultural CR devices require durability and compatibility with soil applications (either under or above soil). In addition, ground soil can require more than simple controlled release features in order to function properly. For example, granular formulation of CR microparticles used in agrochemical applications often need to remain negatively buoyant so that the granules remain in place even when there is run-off during the rainy season. It is also important to protect the polymers from the adverse effects they may encounter when subjected to extreme weather conditions during the dry season, including high levels of ultraviolet radiation caused by prolonged exposure to sunlight. These protective design features are difficult to implement with polymer formulations alone, and therefore may require an additional organic or inorganic “matrix”, to assist in achieving the overall delivery objectives. In this case, the matrix functions as the delivery “vehicle” for the drug microparticles.

The choice of a particular CR system depends on the physical and chemical properties of the substance to be encapsulated and the physical requirements of the polymeric encapsulating material. Encapsulating polymer properties can be controlled by varying the monomer structure, molecular chain length, and the chemical composition. Further control over the polymer system is afforded by the chain flexibility; this too can be varied through the polarity, branching and side chain length^6,7^. The shape, size and degradability of the vehicle used to encapsulate the AI are also important parameters that affect the release profiles. The polymers in these systems may be biodegradable or non-biodegradable. The most commonly used natural polymers are the polysaccharides cellulose, agarose, dextran, alginates, starch, chitosan and proteins including gelatine and albumin^8^. The most frequently used synthetic polymers are polystyrene, polyacrylamide, polymethylacrylate, polyamides, polyesters, polyanhydrides, polyurethanes, amino resins and polycyanoacrylates^3^. Inorganic materials have also been used, including silica, zeolites, inorganic oxides, clays, glass beads, and ceramics^9^.

CR particles include microcapsules, microspheres, coated granules and granular matrices^10,11^. In microcapsules, an AI in the core is surrounded by a shell or membrane, and there may be several cores and several shells. The core can be solid, liquid, gaseous, or a combination. The protective matrix may be an organic or inorganic polymer or even a metal oxide^12^. CR microparticles are cost-effective and straightforward to produce in large quantities, making them ideal for agrochemical applications. Techniques to produce the microcapsule CR systems include spray-drying, spray-cooling, extrusion, freeze-drying, co-crystallization, emulsification, and photopolymerization^13,14^. Emulsification techniques involve dissolving or dispersing the AI in a polymer solution, preparing an oil-in-water emulsion and evaporating the solvent to create the particles after the emulsification^15^. The result is particles of cross-linked polymer with encapsulated AI inside them. The particles can be from 20 nm up to 2000 μm in diameter and can have a spherical shape or an irregular shape. The particles can be composed of one or more polymers. Molecular tailoring, as well as controlling the shape, size and degradation properties of the polymer(s), makes the desired AI release possible^16,17^.

Common substance types or AI’s which can be encapsulated for agricultural uses are nutrients, fertilizers, anti-microbial chemicals and different growth modifiers. Paclobutrazol (PBZ), Flurprimidol, and Uniconazole are all plant growth retardants (PGR) that have similar chemical structures. They inhibit the production of gibberellins (GA) in plants at similar sites in the GA production process^18^. PGRs can be applied to the shoots or to the roots, which allows a broad range of application choices including drenching and foliar sprays. The chosen growth retardant for this research is paclobutrazol which is a triazole derivative. PBZ is a hydrophobic AI that is commercially available both as a dry powder and in a solution. It has been effectively used to induce and manipulate flowering and fruiting in a wide range of crops^19^. PBZ functions as a growth retardant by reducing GA level, increasing cytokinin content, root activity and C: N ratio^20^. It has been characterized as an environmentally stable compound in soil and water environments with a half-life of more than a year^21^. However, it is difficult to detected PBZ residues above quantifiable level (0.01 ppm) in soils and fruits when applied at an optimized rate^22^. The potential of PBZ to contaminate groundwater at optimum concentrations is low; however, the risk of its exposure to aquatic life is high. PBZ is considered moderately hazardous for human beings with remote chance of being toxic^21^.

The advantages that stem from encapsulation of PGRs in CR microparticles include separation of components to avoid possible incompatibilities and protection of different compounds from harmful action of various environmental conditions. Immobilization of sensitive substances can also prolong their efficacy period and help avoid possible overdose for single-shot treatments. One of the most common, convenient, and widely used methods to prepare delivery vehicles for AI is matrix encapsulation of microparticles for sustained delivery of the AI. The matrix encapsulation method is achieved by dispersing CR microparticles in a precursor solution of organic polymers or monomers, and solidifying the suspension into a continuous solid matrix by chemical, physical or ionic cross-linking. Excipients such as inorganic fillers are often added to alter buoyance and provide protective effects from sunlight and other harsh environmental conditions. The products can be made into ribbons, sheets or granules. The entrapped AI-laden microparticles can be liberated and released from the granules by means of controlled biodegradation of the matrix. Alternatively, release of AI from the microparticles can be facilitated by passive diffusion from a non-biodegradable matrix. The choice of a biodegradable or non-degradable matrix will depend on the desired mechanism of AI release: diffusion, matrix erosion, bulk degradation, or combination of diffusion and erosion/bulk degradation^23^.

A number of CR technologies have been developed over the years to accommodate the growing demand for agrochemical devices, including hollow fibers, plastic laminates, paraffin wax, emulsions and microparticles. These systems provide release durations that last from approximately 20 days and up to 100 days^24,25^. The mechanism of release in these systems includes passive diffusion of AI, degradation-mediated liberation of encapsulated AI, and combinations of the two. Yet, prolonging the release profiles of AI beyond 12 months in microparticles and matrix-base delivery systems has been a challenge for the agrochemical industry. Many of the polymeric systems and matrices that have been used are purposely inexpensive, but these lack the control features that can be applied to engineer much longer delivery times beyond 12 months. One possible solution to this problem is to compound the CR features of microparticles and matrices. Controlled AI release from such compounded systems can be delayed by several mechanisms, including diffusion through a rate-controlling membrane, osmosis, ion exchange or coating degradation.

Polymer matrices used in agrochemical dispersal break down by homogenous (bulk) or heterogenous (surface) degradation^26^. Homogenous degradation is most common; hydrolysis usually proceeds by loss of molecular weight^27^. The rate varies with polymer composition and size or shape^28^. Surface degradation rate depends on surface area and geometry; the erosion time is controlled by the radius to thickness ratio rather than the volume^29^. There is no significant change in polymer molecular weight with time. This mechanism requires that surface degradation be faster than water penetration into the matrix^30^. Zero order AI release is obtained with systems such as polyanhydrides or polyorthoesters. The design is simpler because release rates can be controlled by changing thickness and total AI content^31^. The release rate can also be predicted using mathematical modeling, including for different geometries such as degrading slabs, spheres and infinite cylinders^32^. These models must assume that erosion is the rate-limiting step and that AI release occurs from the surface without seepage from the matrix.

In view of the growing demand for multi-year, multi-season CR agrochemical devices, combined with the requirements to develop materials that do not burden the natural environment, it seems valuable in this context to investigate agrochemical uses for biodegradable polymers which are normally used for biomedical purposes. Accordingly, our hypothesis asserts that by combining beads of alginate hydrogel and PCL microspheres in a single system, we can create a multifunctional slow-release device. We further hypothesize that by controlling the physical parameters of the capsule, namely the size, we will be able to control the release rate of AI from the device for multi-season release in a single treatment. The overall goal of the proposed project was to engineer carriers to release the PBZ over the course of a couple of rainy seasons after their dispersal on the soil. The formulations were prepared by encapsulating the PBZ in microparticles of PCL and encapsulating the PCL-PBZ microparticles in alginate/clay beads that are freeze-dried into granules. The PCL-PBZ microparticles were also designed to undergo controlled degradation and subsequent release of PBZ based on a pre-determined release kinetics. The experimental plan included a set of detailed studies aimed at refining the control over the mean diameter and polydispersity of the PCZ-PBZ microparticles for subsequent use as a controlled delivery system. Additional control features include the composition of the alginate/clay beads. Scanning electron microscopy (SEM) imaging was performed in order to visualize the integrity and homogeneity of the beads and the microparticles. The effect of different physical parameters of the microparticles and beads on the release rates of the AI into water was evaluated. In order to test the effect of PBZ release on plants, a single formulation was chosen and tested in accelerated washing experiments and subsequent bioassays. A model system based on the oatmeal plant was used for bio-assaying controlled release upon exposure to multiple simulated rainy seasons. Additionally, the chosen formulation was scattered onto large outdoor fields and preliminary field experiments were photographed for two rainy seasons, while biomass values were quantified by image analysis.

## Materials and methods

### PCL microparticle preparation

PCL microparticles were prepared by oil in water (o/w) solvent evaporation method^33^. The PCL polymer (10% w/v, average Mn 80,000 Da, Aldrich) was mixed in dichloromethane (DCM) (Anhydrous, Aldrich) with 8% (w/v) of Paclobutrazol (PBZ) (Sigma-Aldrich). This solution was then placed by bolus injection into 1% (w/v) polyvinyl alcohol (PVA) (Mowiol 4–88, average Mw 31,000, Aldrich) in water, such that the final concentration was 2% (v/v) DCM in PVA/water. The PVA acts as an emulsifier to the PCL in water, thus helping to create an emulsion when the mixture is vigorously homogenized using a high-power homogenizer (IKA Ultra Turrax, T18 Digital, IKA-Werke GmbH & Co. KG, Staufen, Germany). After 5 min of homogenization (6600 to 20,600 RPM), the emulsion was kept overnight on a magnetic stirrer to allow evaporation of the DCM. The hardened microparticles were collected by centrifugation at 3250 rpm for 6 min in order to remove the PVA solution. Finally, the microparticles were washed with deionized water and lyophilized overnight. The microparticles in their final dry form were then characterized or used for controlled release studies. Characterization of the microparticles included size determination using a laser diffraction method (Mastersizer 3000 with Hydro MV unite, Malvern Panalytical, Malvern, United Kingdom) and imaging using a scanning electron microscope (SEM) (Quanta 200, FET) to visualize surface features and homogeneity, as further described below.

### PLGA microparticle preparation

Poly(lactic-co-glycolic acid) (PLGA) microparticles were prepared using an emulsification method described elsewhere^34^. Briefly, 10% (w/v) PLGA (lactide:glycolide [75:25]; mol.wt. 66,000–107,000; Aldrich) and 10% (w/v) Polylactic acid (PLA) were mixed in DCM (Anhydrous, Aldrich) with 4% (w/v) of PBZ (Sigma-Aldrich). This solution was then placed by bolus injection into 1% (w/v) PVA (Mowiol 4–88, average Mw 31,000, Aldrich) in water, such that the final concentration was 1% (v/v) DCM in PVA/water. The emulsion was kept overnight on a magnetic stirrer to allow evaporation of the DCM. The hardened microparticles were collected by centrifugation at 3250 rpm for 6 min in order to remove the PVA solution. Finally, the microparticles were washed with deionized water and lyophilized overnight. The microparticles in their final dry form were then characterized for size determination using a laser diffraction method (Mastersizer 3000, Malvern Panalytical) and imaged using SEM (Quanta 200, FET).

### Alginate bead preparation

Alginate (Alginic acid sodium salt powder, Aldrich) was dissolved in water to create a 1.2% solution (w/v). After the alginate reached complete dissolution under constant stirring, clay mineral (Montmorillonite, MT) K10 powder (Aldrich) was added as filler (~ 1.5%, w/v) in order to increase bead stability^35,36^. In addition, the PCL microparticles were added to the alginate solution at a 1:1 ratio of dry weight (PCL: alginate). The mixture of alginate-MT-PCL-PBZ was thoroughly homogenized and filtered through a 600 µm sieve. Homogenization and filtration allowed extrusion through a small orifice of an encapsulation machine (Buchi B-395 Pro, Buchi, Flawil, Switzerland) and into a 1 M CaCl_2_ cross-linking bath below. In order to achieve desired size (~ 1 mm), the Buchi encapsulation machine was fitted with am extrusion nozzle having a characteristic size of 750 µm. The parameters used with this extrusion nozzle were 229 mbar of pressure, a vibration frequency of 80 Hz, a vibration amplitude of 5 µm and an electrode charge of 250 V. The solution was extruded and allowed to break away from the nozzle in droplet form by applying mechanical perturbation to the nozzle with a piezoelectric element. Upon contact with the cross-linking solution, the droplets formed alginate beads of a spherical geometry and diameter of ~ 1500 µm that were further characterized as detailed below.

### Characterization of size and surface characteristics

#### Laser scattering for PCL microparticle size

Size distribution of the PCL microparticles was determined by a laser diffraction method. Size analysis was performed with Mastersizer 3000 (Malvern Panalytical). The microparticles were suspended in double deionized water (DDW) and dripped into the test chamber until the proper turbidity value in the range of 5–8% was reached, as indicated by the Mastersizer 3000. Each sample average was represented by five independent measurements from at least 6 specimens.

### Surface and size characterization using SEM

The size and porosity of the alginate beads and PCL microparticles was assessed directly from scanning electron micrographs. In order to enable imaging of PCL microparticles by SEM, the samples were first coated with gold (Au)/palladium (Pd) as described elsewhere^37^. The coating was applied using a sputter coating machine (SEM coating unit E5150, Polaron Equipment LTD). A similar coating was applied for visualization of the alginate beads. The PCL microparticle and alginate bead size measurements were conducted using ImageJ image processing tools. Each sample average was determined from at least 6 specimens; each specimen was represented by five independent ImageJ measurements from the SEM images. Bead porosity was visually assessed from the scanning electron micrographs.

### Testing AI release in water

The formulated beads were incubated in water and the rate of release of the PBZ from the beads was determined periodically. Each bead sample, (44 mg, 10% PBZ %w/w), was suspended in 125 ml distilled water. A portion of the sample volume (1 ml) was periodically taken for analysis by mass spectrometry (MS) combined with High Pressure Liquid Chromatography (HPLC) (HPLC–MS). The periodic removal of sample volume was compensated by adding 1 ml of fresh distilled water to each sample, so that the sample volume always remains 125 ml. The cumulative release of AI was quantified and reported as a function of the incubation time to provide the release kinetics of the system. Normalizing the AI release data required knowing the total amount of AI remaining in the alginate beads at the termination of the release experiment. The remaining AI was measured by first dissolving the alginate using a divalent cation chelator. The dissolution was performed by placing the alginate beads into a solution of 100 mM tri-sodium citrate as the calcium chelator. The beads were incubated overnight in this solution using a water bath heated to 37 °C. The quantity of remaining AI in the samples of the dissolved beads, as measured by HLPC-MS, was added to the cumulative released AI values at the termination of the experiment, in order to determine the total amount of AI in the beads for each sample. The transient release data of each sample was then normalized by the respective total AI value for that sample.

The protocols for quantification of PBZ were developed by the department of chemistry, Technion—Israel Institute of Technology. Samples were filtered and then injected first into an HPLC (Dionex, Ultimate 300) for initial peak separation. HPLC separation was performed with Phenomenex omega column (C18, 1.6 µm, 50 mm × 2.1 mm). Standard conditions for HPLC are described in Table 1.

**Table 1:** HPLC procedure for sample peak separation.

Retention (min)	Flow (ml/min)	A*%	B**%
0	**0.2**	**10**	**90**
5	**0.2**	**90**	**10**
8	**0.2**	**90**	**10**
9	**0.2**	**10**	**90**
13	**0.2**	**10**	**90**

*Acetonitrile + 0.1% Tri-fluolroacetic acid; **Water + 0.1% Tri-fluolroacetic acid.

After the HPLC separation, each peak was quantified by MS (Maxis Impact, Bruker). PBZ has a molar mass of 294 g/mol and flight time of approximately 5–8 min. Standard conditions for MS were as follows: ESI: Electron positive; Capillary: 4000 V; Nebulizer: 1 bar; Dry gas: 8 L/min; Dry temperature: 2000C. For each group of samples delivered for testing, four calibration samples were prepared in order to create a calibration curve: 3 ppm, 9 ppm, 12 ppm, 18 ppm (Supplementary Fig. 1). Using the calibration curve, peak heights were converted to AI concentration in ppm (Supplementary Fig. 2).

### Biological assays

#### Oatmeal growth assay

**Figure 1:**
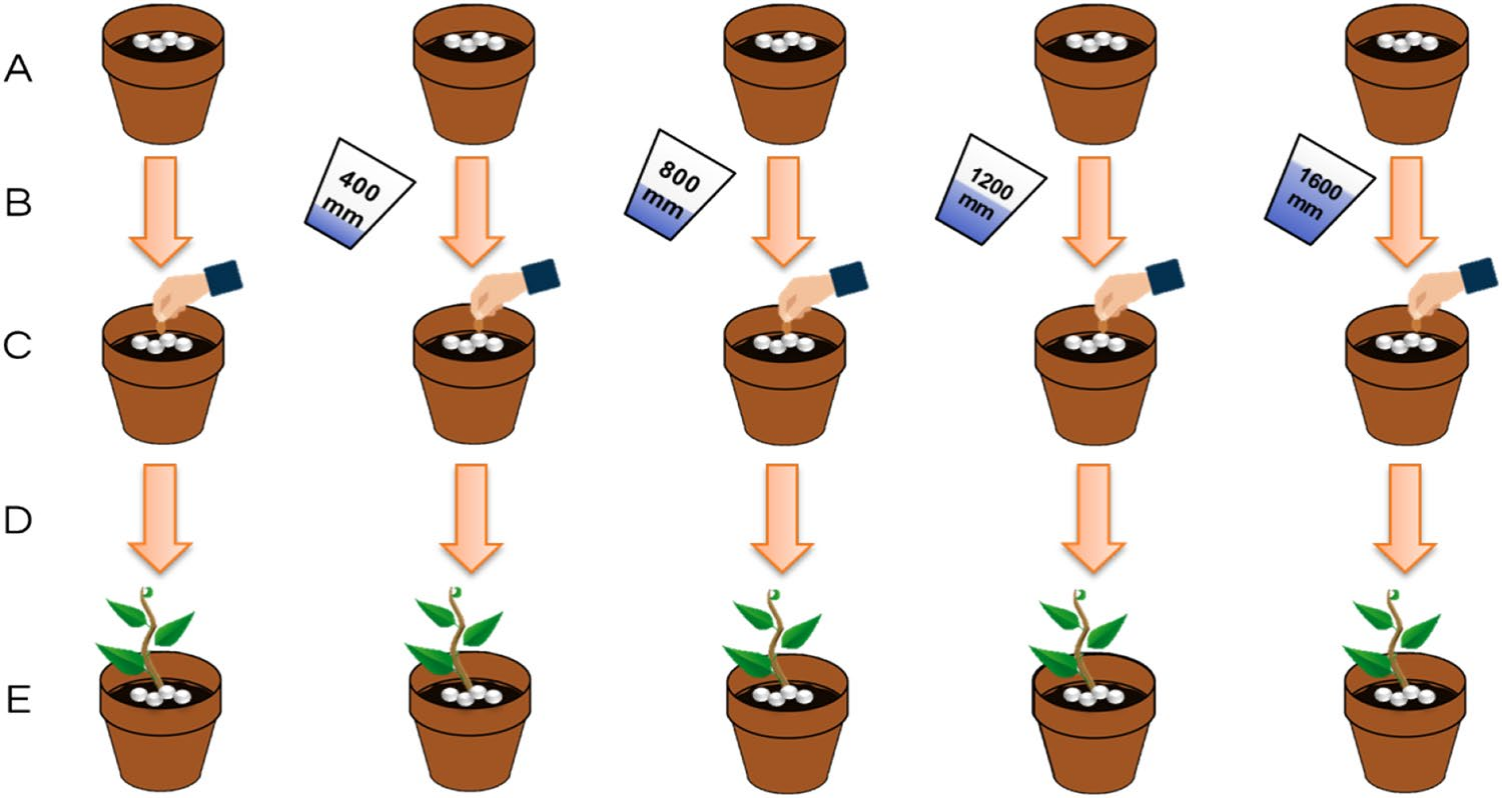
Overview on the process of accelerated washing experiment. Dispersing beads (**A**); washing (**B**); Seeding (**C**); germination (**D**); and Measuring (**E**).

Controlled release of PBZ from beads—under conditions that are intended to simulate multiple rainy seasons—involved the preconditioning of the beads in wash-water volumes that correspond to a single rainy season, or multiple rainy seasons (Fig. 1). Sieves filled with soil were used to allow the particles to undergo continuous washing with the simulated rainwater. One year of rain was represented by 400 mm of wash-water on the beads. Each rainy season was accelerated to 2 weeks, with eight wetting periods, 5-min each, occurring consecutively within a 24-h timeframe (continuously for the two-week timeframe). Each washing period was followed by drying of the beads and sieves in 50 °C for 24 h in order to simulate the dry season (maximum of 4 alternate wet–dry periods). The preconditioned beads were then collected and scattered on top of stainless-steel sieves (2-mm cut-off) filled with oatmeal plant seeds in soil samples collected from the Sorek region in Israel. A strain of Sia oatmeal was used as a model plant to test if the PBZ that remained in the beads after their respective washes—and was subsequently released into the growth soil—affected the growth rate of the plant. Seeds were allowed 14 days of germination and then visually inspected for sprout height (measured manually with a ruler). In addition, sieves were photographed, and leaf area was measured using image analysis (ImageJ software). In addition to measuring oatmeal growth, we sampled the collected water which were washed from the oatmeal-bearing sieves. Samples were taken at every 200 mm of water. Cumulative release of PBZ was quantified by methods of Mass spectrometry (MS) combined with High Pressure Liquid Chromatography (HPLC) as detailed below.

### Preliminary field experiments

Beads were scattered manually in a field located south of Kiryat-Gat, Israel. The beads which were scattered contained 17% (%w/w) of PCL microparticles, 23% (%w/w) MT as filler and 10% (%w/w) of PBZ (70% in PCL, 30% in alginate). The finale dosage on the surface was 2 g PBZ/m^2^. The dimensions of each scattered plot were 3 × 3 m (9m^2^). Plots were observed each winter during 2 consecutive years. In addition, the experiment was photographed by an unmanned aerial vehicle (UAV). The resulting photographs were analyzed by calculating the variance of RGB values in the aerial photo in comparison to areas which were not treated (Eq. 1). Our quantification method assumes that viable plants have higher variance of RGB values, as compared to non-viable plants and soil. Other vegetation indices such as TGI and VARI might be considered when analyzing the UAV photos. Results were normalized to untreated vegetation as described elsewhere^38^.

1 $$\% Vegetation = \frac{{Stan dard\,deviation\cdot\,Treated({{Red}} ,Green,Blue)}}{{Standard\,deviation\cdot Not\,Treated({{Red}} ,Green,Blue)}}$$

**Equation ****1**: Vegetation index for quantification of biomass on treated plots.

### Statistical analysis

Results are reported as mean ± standard deviation (SD). Comparison between multiple treatments were performed using ANOVA (analysis of variance). A Bonferroni's correction was used. A p value of less than 0.05 was set as a significance threshold. GraphPad prism analysis software (Version 5.01, GraphPad Software Inc., CA, USA) was used for this analysis.

## Results

### Alginate beads

**Figure 2:**
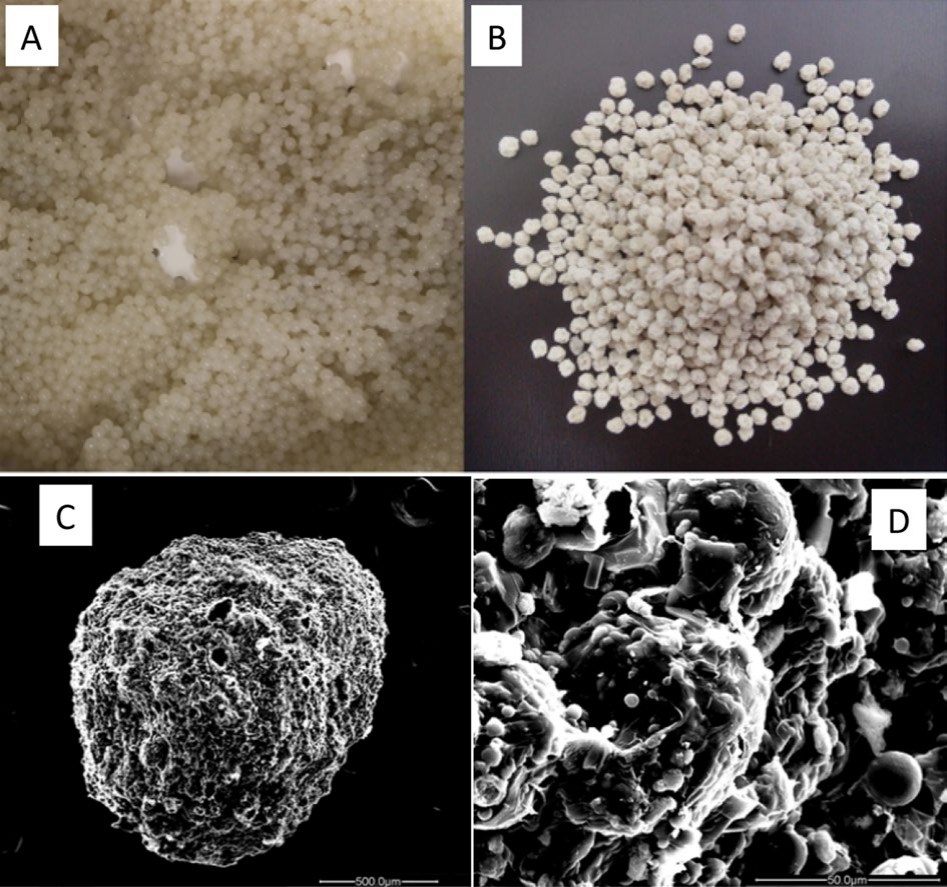
Alginate/clay beads (i.e., granules) containing PCL-PBZ microparticles. The granules are shown before (**A**) and after (**B**) lyophilization. SEM images of the beads at low magnification (**C**, scale = 500 µm) and at high magnification (**D**, scale bar = 50 µm).

Alginate/ clay mineral (MT) beads containing PCL/PZB microparticles had a rough surface appearance (Fig. 2). The size of the lyophilized beads was measured by a caliper to be 1.52 ± 0.15 mm in diameter. This size was a function of the Buchi encapsulator nozel size and flow/vibration settings. The sieves were process through a sieve with a characteristic size of 1–1.4 mm. During the preparation of the beads, the exact volume of the dissolved alginate with 1.2% PCL and 1.7%MT were extruded and their weight before and after freeze-drying was measured. Lyophilization resulted in loss of 92% of original water content. The final PCL concentration was 17% (% dry weight).

### PCL microparticles

**Figure 3:**
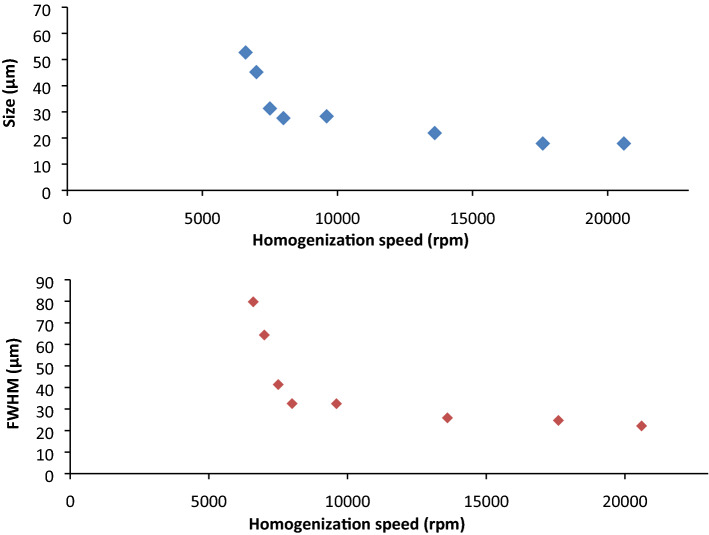
(**A**) Effect of homogenization speed on the size of PCL microparticles (with 1% PVA). (**B**) Full width half maximum (FWHM) of different PCL microparticles as a function of homogenization speed.

Different batches of PCL microparticles were prepared using various speed settings on the dispersing instrument, ranging from 6600 to 20,600 RPM. The microparticle size distribution as a function of homogenization speed was characterized (Fig. 3A). Constant emulsion composition of 1% PVA was maintained for all homogenization speeds. In order to account for the amount of dispersion in SLS measurements, the full width half maximum (FWHM) data was extracted using OriginLab software (Fig. 3B). FWHM is the width of the distribution function curve measured between those points on the y-axis which are half the maximum amplitude. The analysis was based on size distribution of particles derived from Mastersizer measurements.

**Figure 4:**
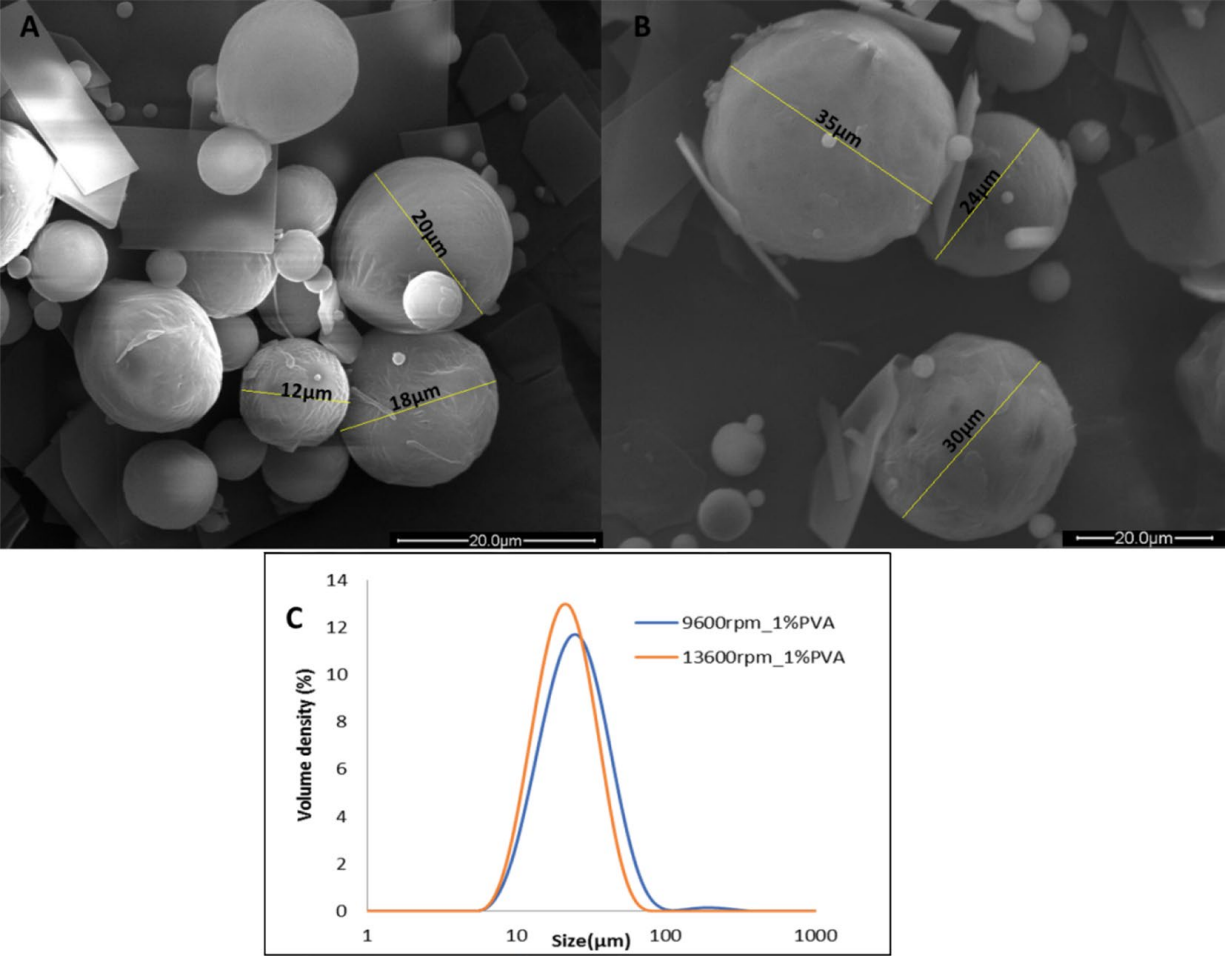
SEM images of PCL microparticles produced with two different homogenization speeds: (**A**) 9600 rpm and (**B**) 13,600 rpm. (**C**) Mastersizer data of the mean microparticle size with two different homogenization speeds.

Characterization of the microparticles included SEM imaging to visualize the surface features and homogeneity of the specimens. Figure 4 shows the SEM images of PCL microparticles prepared using two different homogenization speeds (in revolutions per minute, rpm) along with the distribution chart from the Mastersizer scan. The Mastersizer results show the mean microparticle size and the polydispersity, as a function of homogenization speed. When measuring particle size by SEM, only a small fraction of microparticles can be seen, whereas in the laser diffraction, the whole size scale is represented. Consequently, results of size measurements using laser diffraction matched those measured by SEM.

### PLGA microparticles

**Figure 5:**
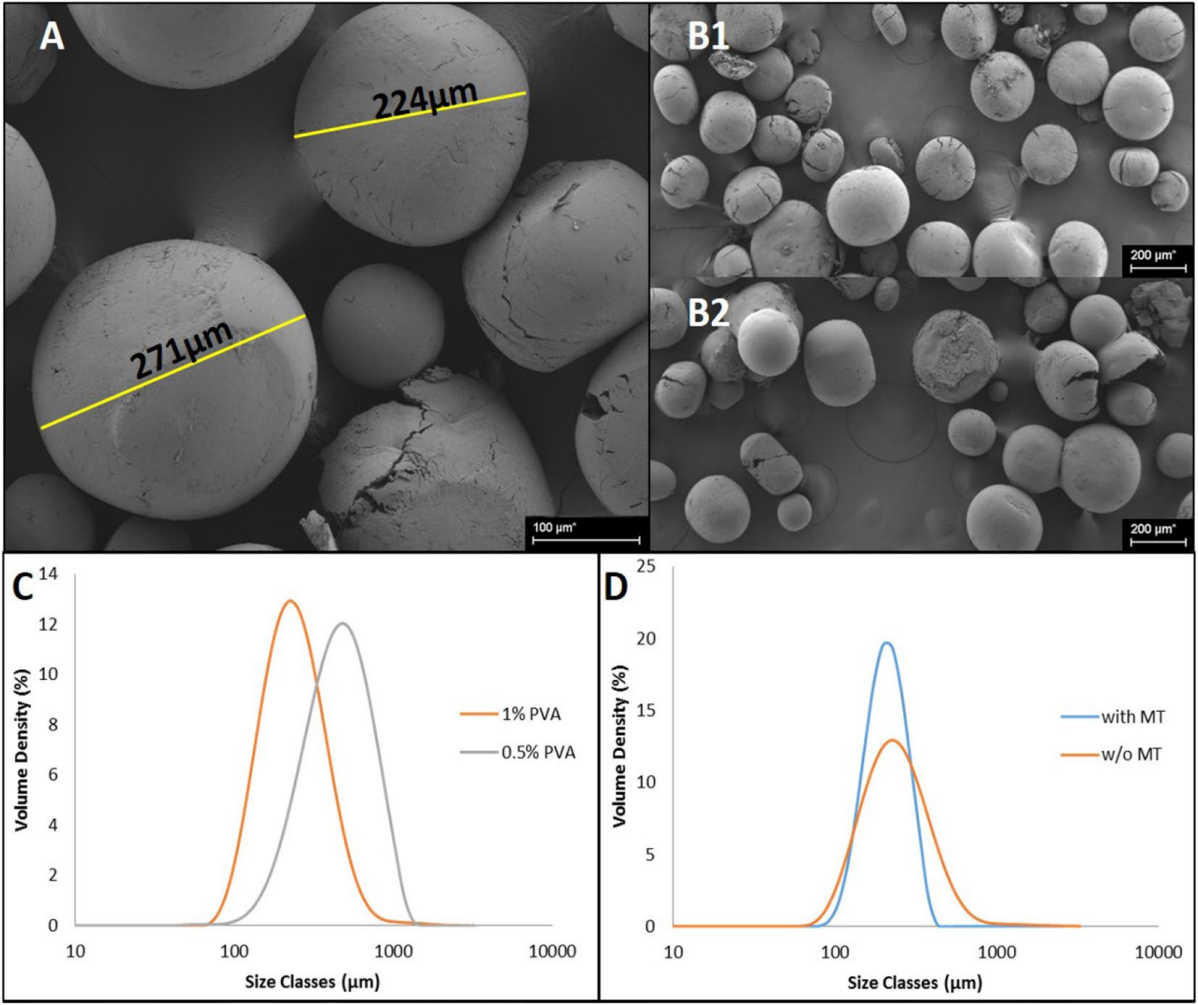
Preliminary experiments with PLGA-PBZ microparticles, with MT clay and without MT clay. SEM images of the PLGA-PBZ microparticles, with (**B2**) and without clay (**A**, **B1**). Mastersizer results showing PLGA-PBZ particle size distribution with two PVA concentrations (**C**). Mastersizer results showing PLGA-PBZ particle size distribution with and without MT clay (**D**).

Preliminary results with the PLGA suggested that this system can also be applied with the emulsion solvent evaporation technique to prepare PBZ microparticles, albeit with much larger microparticles being produced (~ 250 µm) (Fig. 5). The larger size of the PLGA microparticles which were produced by the emulsion method was a major limitation for subsequent use with the Buchi encapsulator nozzle. Consequently, alginate beads embedded with PLGA microparticels were not pursued further because of this size limitation.

### Emulsion composition

**Figure 6:**
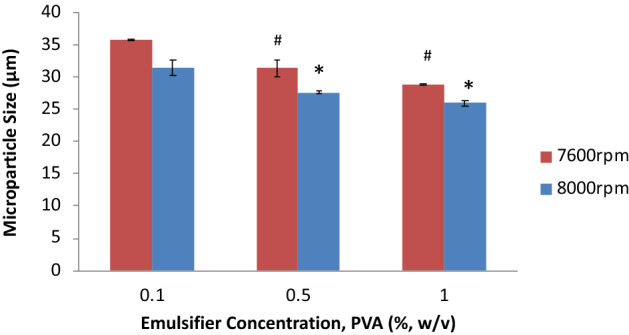
Effect of PVA emulsifier concentration on the size of PCL microparticles homogenized at 7600 RPM. The different emulsifier concentrations caused a statistically significant difference in microparticle size (ANOVA, n = 5, *p* < 0.05); #, * indicate statistically significant difference compared to 0.1% PVA concentration treatment, for 7600 rpm and 8000 rpm data, respectively (n = 5, *p* < 0.05).

Different concentrations of PVA were combined with two homogenization speeds. The concentration of emulsifier was limited by the level of solubility of PVA in water. Emulsifier concentration of 2% resulted in a solution with limited homogeneity. On the other hand, under PVA concentrations lower than 0.1%, the emulsion was not stable, resulting in sedimentation and coalescence of polymer droplets. Results show a moderate reduction in the mean diameter of the microparticles as the concentration of the emulsifier was increased from 0.1 to 1% (w/v) (Fig. 6). The reduction in size was more significant for 7600 rpm than for 8000 rpm.

### Release experiments

**Figure 7:**
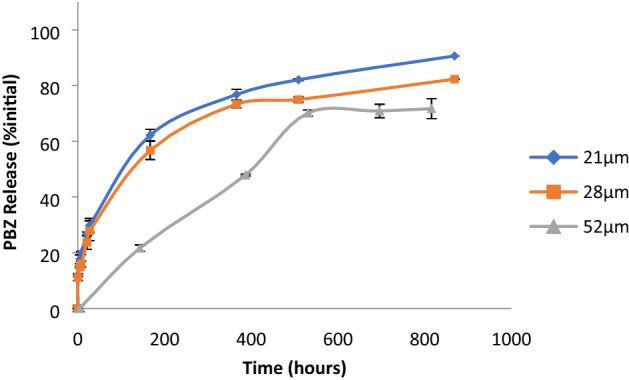
Effect of PCL microbeads size embedded in alginate beads on release of AI from alginate beads.

Alginate beads containing 1.7% MT, 1.2% PCL and 7% PBZ (in PCL microparticles) were suspended in water. During the first 27 h after submersion, samples were taken with high frequency (i.e., every 0.5–2 h). After the initial 27 h period, the time in between sample measurements was extended to ~ 200 h. In order to test the effect of embedded PCL microparticle size on release from alginate beads, two batches of PCL microparticles were synthesized using two different homogenization speeds (9600 rpm and 13,600 rpm). According to Mastersizer measurements, the above homogenization speeds yield microparticles of 21 µm and 28 µm, respectively. The release results indicate the effect of larger size on release rate. Percent of release from beads of 21 µm after 869 h (36 days) was ~ 10% higher than release from beads of 28 µm (Fig. 7).

### Oatmeal growth assay

**Figure 8:**
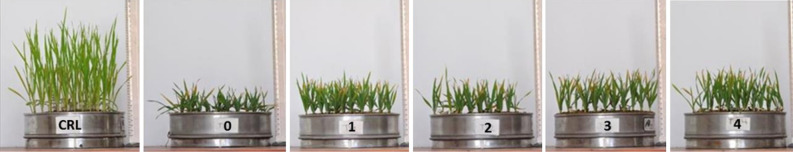
Oatmeal sprouts grown for identical amount of time on sieves with soil and Alginate/PCL granules which were washed with amounts of water that represent 0 to 5 rainy seasons. Control contains no granules (CRL); each treatment is labelled with the number of rainy seasons of pre-washing that were used in the experiment.

**Figure 9:**
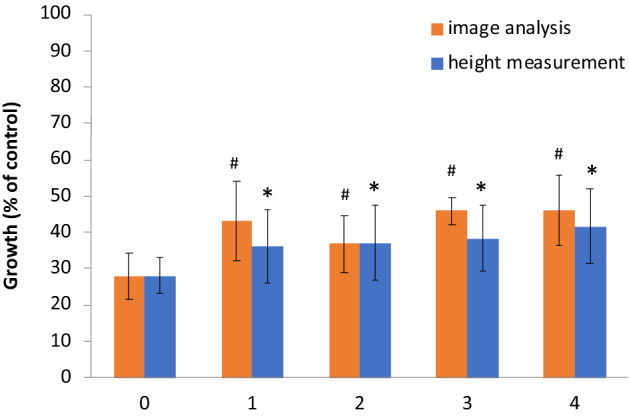
Growth of oatmeal in soil treated with Alginate/PCL beads (1.9 g/m^2^ PBZ) pre-washed with amounts of water that represents 0 to 4 rainy seasons. The data is compared to a control treatment without granules. The growth is analyzed using height measurement and image analysis. All treated samples demonstrated a statistically significant growth retardation when compared to the control group (n = 6, *p* < 0.01); #, * indicate statistically significant difference compared to season 0 treatment for image analysis.

Oatmeal seeds were grown in the presence of the pre-washed alginate granules containing 17% PCL, 23% MT filler and 10% PBZ (70% in PCL, 30% in alginate). The pre-washing was performed with an amount of water that represents from 0 to 4 rainy seasons and compared to control samples grown without granules. The effect of PBZ released from the pre-washed alginate granules on oatmeal sprouts was then measured two weeks after seeding using two different methods: a height measurement and an image analysis algorithm of the oatmeal sprouts. The quantitative growth results in each measurement method were represented as the percent of control. Results show a relative reduction in growth compared for controls (without granules) for all pre-washing periods (i.e., 0 to 4 seasons) (Fig. 8). The quantification of the oatmeal growth, as measured by the two techniques, showed that most of the growth retardation was observed for the treatment without washing (i.e. year 0) (Fig. 9). For each subsequent pre-washing cycle that represents an additional rainy season, the results indicated a slight increase in oatmeal growth (41–46% growth after 4 cycles, relative to control without beads). Results of growth measurement were similar for both methods (height measurements and image analysis) (data not shown).

### Preliminary field experiment

**Figure 10:**
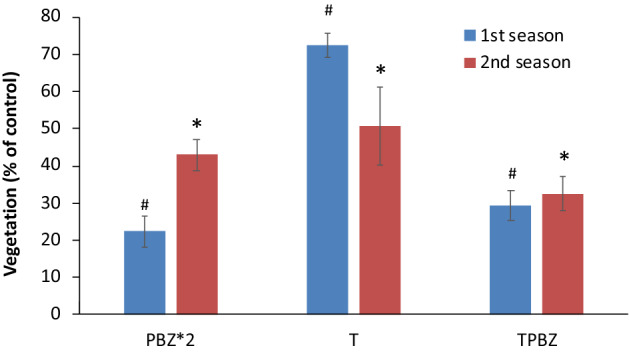
Amount of vegetation relative to control in treated plots as measured by extracting values of RGB variations using image analysis of the plots. PBZ*2 is 2 g of PBZ/m^2^ (neat); **T** is 2 g of PBZ/m^2^ (in granules); **TPBZ** is 2 g PBZ/m^2^ (in granules) with 1 g PBZ/m^2^ (neat). The different treatments demonstrated a statistically significant growth retardation (ANOVA, n = 3, *p* < 0.01); #, * indicate statistically significant difference compared to season 0 (control) for season 1 and season 2 measurement data, respectively (n = 3, *p* < 0.01).

Granules of alginate containing 23% (%w/w) MT as filler, 17% (%w/w) PCL microparticles and 10% (%w/w) PBZ were manually dispersed on designated plots of land. The beads contained 2 g of PBZ for each 1 m^2^ of covered ground. Along with alginate granules, control plots containing neat PBZ were dispersed in two different concentrations: 1 g of PBZ/m^2^ and 2 g of PBZ/m^2^. The plots were observed for two seasons. Values of RGB variation were extracted from unmanned aerial vehicle (UAV) photos which were taken during the winter season in the first year and second year of the field experiment (Supplementary Figs. 3 and 4). Analysis of RGB variation values was performed for both seasons for all plots and normalized to untreated terrain which constitutes the negative control in the experiment (Fig. 10).

Analysis result for two seasons resulted in different trends for each treatment. For the neat 2 g of PBZ/m^2^ treatment (PBZ*2), the lowest biomass for the first season (22% on average) was recorded. The second season was about two times higher than the first season for this treatment (42% in average). For the granular treatment containing 2 g of PBZ/m^2^ (T), the highest biomass for both first and second season were recorded (72% and 50%, respectively). A decline of 30% in the second season relative to the first season was evident in this treatment. Finally, for the combined granule and neat PBZ treatment, TPBZ (i.e., 2 g PBZ/m^2^ in granules and 1 g neat PBZ/m^2^) both seasons showed relatively similar vegetative growth (29–32% on average).

## Discussion

The aim of this work was to define a formulation of alginate-MT-PCL MPs that contain at least 10% (%w/w) PBZ and maintain a release profile that can accommodate multi-year rain cycles. Alginate and PCL have different rates of degradation, but together they can form a pattern of release for prolonged periods of time. The alginate was designed as a stable carrier matrix for the PCL microparticles. The PCL microparticle size and formulation was engineered to provide the sustained release pattern for the controlled growth retardation over multiple seasons. Consequently, other polymers were tested as encapsulants, specifically as alternatives to the PCL. These include PLGA or slow degrading polyurethanes (PU). The advantage of PLGA is the ability to encapsulate the PBZ in a much faster degrading polymer system, for enhanced release kinetic based on more rapid hydrolytic breakdown. In preliminary experiments PLGA-PBZ microparticles demonstrated the mean diameter of 250 µm without clay addition (Fig. 5A). Mastersizer measurements confirmed this mean diameter and demonstrated how one can control diameter using PVA (Fig. 5C). Addition of montmorillonite clay to the PLGA was also examined as a means of controlling diameter of the microparticles, with less significant outcomes (Fig. 5D). The disadvantage of the PLGA is the cost of the raw material, when compared to PCL or PU.

The concentration of AI inside the granules is an important factor which is utilized for obtaining the desired AI concentration in the context of this study. We are able to control the amount of AI in the beads when it is necessary to adjust the amount of PBZ to a particular application. It is understandable that different types of vegetation will require a different dosage of PBZ to achieve the desired outcome. For instance, perennial plants require higher dosage than annuals. The amount of PBZ loaded into the compound bead (alginate with PCL microbeads) is restricted by the chemical properties of polymers and the synthesis parameters. In order to allow sufficient growth regulation by PBZ for long periods of time, the %weight of PBZ in the capsule should be high enough to give an appropriate dose each year. On the other hand, given first order AI release (from alginate matrix), the higher the AI loading, the higher the release rate. Balancing of loading and release rate can be achieved by adjustment of AI allocation in alginate or PCL microbeads. Accordingly, it is also possible to apply a stochastic approach in order to manipulate the distribution function of PBZ in time. For example, one can try to control the parameters of the granules so as to manipulate the distribution function to achieve 5-years full width at half maximum (FWHM). Thus, we can achieve the desired release of PBZ distributed over 5 years by producing granules containing different amounts of PBZ and having different diameters. Higher refinement of the controlled release system can be achieved by developing a polymeric coating method^39^.

Encapsulation of the AI inside the alginate granules constitutes another feature of controlled release in this system. When the AI is located solely in PCL microcapsules, this results in a delay in PBZ release, which can be overcome by the incorporation of the AI in the alginate. Depending on extent of delayed release from the PCL microparticles, the release is compensated with initial burst of AI from the alginate. To accomplish this, the AI is concentrated in the alginate matrix (suspended during dissolution of alginate in water), and the expected release rate is faster because the alginate is a hydrophilic and biodegradable polymer which degrades faster than PCL.

The CR granules, when tested for AI release while submersed in water, showed that one can control the release rate from the system using the PCL microbead size. Increasing PCL microbead size had a negative effect on release from beads. Beyond the release experiments in water, irrigation cycle experiments were conducted in order to improve resemblance to field conditions where water is flowing rather than stationary, and a rainy period is followed by a dry period. Rainy season simulations resulted in gradual accumulation of PBZ in the washing water which corresponds to our expectations and conferred our initial hypothesis that slow release for prolonged periods of time is possible using this approach. Results of this bioassay showed presence of PBZ for all washing periods, which has implications for possible long-term release results. We would expect strong growth retardation in the first year and then a rise in growth with subsequent rainy cycles. Moreover, high dosage of readily available PBZ can have an adverse effect on germination levels (results not shown).

Preliminary field experiment conducted over two rainy seasons were provided some insight into the performance of the CR system. Although not fully conclusive to our expectations, these experiments indicated that the combined treatment of readily available PBZ within the Alginate particles and the PZB in the PCL achieved the most promising results. Only for this combined treatment, the level of vegetation remained stable for two consecutive seasons. The limitations of this preliminary experiment include the insufficient amount of repetitions and damage which was caused to the scattered plots over the course of two years. We also chose variability of RGB as a vegetation index in order to quantify the level of effect each treatment had on the plants. Other vegetation indices, which are based on visible spectrum, might be used instead. However, values extracted using TGI and VARI indices did not accurately represent the observed growth retardation caused by PBZ (results not shown). The results from the field experiments show a favorable trend of the long-term effects, yet additional seasons of observations are necessary to determine the full effect of beads in natural conditions.

## Conclusions

The current study shows the potential use of common biomedical polymers for agricultural purposes. We were able to create a compound capsule with two different biodegradable polymers, including the matrix (alginate) and the embedded microparticles (PCL) that release the AI. The combination of alginate with PCL allowed us to refine our control over the release of PZB. Accelerated tests and preliminary field experiments showed positive results regarding the ability of the developed capsules to release PBZ for prolonged periods of time, including multiple rainy seasons.

## Supplementary Information

Supplementary Figures.
