# Supplementary material for: Alginate hydrogel beads embedded with drug-bearing polycaprolactone microspheres for sustained release of paclobutrazol
Source: Sci Rep. 2021 May 25;11:10877. doi: 10.1038/s41598-021-90338-9 (PMC8149846; doi:10.1038/s41598-021-90338-9)
Supplement: Supplementary file 1 — Supplementary Figures. [file 41598_2021_90338_MOESM1_ESM.docx]

*Supplementary Figure 1: A calibration sample having a concretion of 12ppm AI was measured by MS. The signal area for the sample was applied to prepare a calibration curve used to determine the AI concentration of the various experimental samples.*

*Supplementary Figure 2: Four calibration samples were used in order to create a calibration curve: 3ppm, 9ppm, 12ppm, 18ppm. To prepare the calibration curve, the signal area for each sample was used to calculate the AI concentration in ppm.*

Supplementary Figure 3: Land plots of scattered alginate+PCL beads photographed by UAV, first season of observation. Each row represents one repetition. Each column represents dosage of PBZ and mode of application.

Supplementary Figure 4: Land plots of scattered alginate+PCL beads photographed by UAV, second season of observation. Each row represents one repetition. Each column represents dosage of PBZ and mode of application.
